# Expansion of the Phenotypic Spectrum of MNGIE: Lipodystrophy and Metabolic Alterations Associated with a p.Arg393_Val400dup TYMP Variant

**DOI:** 10.3390/ijms26199751

**Published:** 2025-10-07

**Authors:** Donatella Gilio, Caterina Pelosini, Silvia Magno, Jacopo Maria Venanzi, Marta Daniotti, Melania Paoli, Lavinia Palladino, Maria Rita Sessa, Franco Ricci, Elena Procopio, Giovanni Ceccarini, Ferruccio Santini

**Affiliations:** 1 Obesity and Lipodystrophy Center, Endocrinology Unit, University Hospital of Pisa, 56124 Pisa, Italy; donatella.gilio@phd.unipi.it (D.G.); silviamagno9@gmail.com (S.M.); l.palladino@studenti.unipi.it (L.P.); giovanni.ceccarini@unipi.it (G.C.); 2 Chemistry and Endocrinology Laboratory, University Hospital of Pisa, 56124 Pisa, Italy; caterina.pelosini@ao-pisa.toscana.it (C.P.); melania.paoli@ao-pisa.toscana.it (M.P.); m.sessa@ao-pisa.toscana.it (M.R.S.); 3 Metabolic and Neuromuscular Unit, Meyer Children’s Hospital IRCCS, 50139 Florence, Italy; jacopomaria.venanzi@meyer.it (J.M.V.); marta.daniotti@meyer.it (M.D.); elena.procopio@meyer.it (E.P.); 4 Diabetology and Endocrinology Unit, Meyer Children’s Hospital IRCCS, 50139 Florence, Italy; franco.ricci@meyer.it

**Keywords:** lipodystrophy, *TYMP* mutation, MNGIE, encephalomyopathy, thymidine phosphorylase, leptin

## Abstract

Mitochondrial neurogastrointestinal encephalomyopathy (MNGIE) is a rare autosomal recessive disorder caused by mutations in the *TYMP* gene, typically characterized by severe and progressive gastrointestinal and neurological manifestations. Recent reports have identified a subset of patients presenting with generalized lipodystrophy and metabolic abnormalities, suggesting that adipose tissue involvement may be an underrecognized feature of the disease. Herein, we report the case of a 16-year-old female carrying a previously described homozygous *TYMP* variant (c.1178_1201dup; p.Arg393_Val400dup), who presented during adolescence with generalized lipodystrophy, insulin resistance, hypertriglyceridemia, hepatic steatosis, and other metabolic complications. At diagnosis, she exhibited no overt neurological or gastrointestinal symptoms; however, electroneurography revealed subclinical peripheral neuropathy. This case broadens the phenotypic spectrum of TYMP-related disease by documenting a lipodystrophic and metabolic presentation associated with the p.Arg393_Val400dup variant. While *TYMP* mutations have been linked to lipodystrophy in rare cases, this specific variant had previously been reported only in the context of classical MNGIE, with no documented evidence of adipose tissue or metabolic derangement. Our findings highlight the importance of considering *TYMP* involvement in the differential diagnosis of atypical lipodystrophy syndromes, particularly when features suggest underlying mitochondrial dysfunction.

## 1. Introduction

Mitochondrial neurogastrointestinal encephalomyopathy (MNGIE) is an ultrarare autosomal recessive disorder caused by loss-of-function mutations in the *TYMP* gene, which encodes for thymidine phosphorylase (TP) [1]. Deficiency of this enzyme leads to systemic accumulation of thymidine and 2′-deoxyuridine, resulting in mitochondrial DNA (mtDNA) instability, depletion, and subsequent mitochondrial dysfunction [2,3]. Clinically, MNGIE presents as a multisystem disorder, most notably involving the gastrointestinal and nervous systems with severe and progressive symptoms [4]. Lipodystrophies are a rare and heterogeneous group of disorders characterized by the absence of adipose tissue, associated with potentially severe metabolic complications, including insulin resistance, diabetes, hypertriglyceridemia, and hepatic steatosis [5,6]. Among congenital forms of lipodystrophy, those caused by mitochondrial dysfunction, although rare, are being increasingly recognized [7,8,9,10,11,12,13].

In recent years, a few patients with MNGIE have been reported to exhibit generalized lipodystrophy with prominent metabolic alterations, raising the possibility that adipose tissue involvement may be an underappreciated component of TYMP-related disease [14]. We report the clinical course of a lipodystrophic patient with MNGIE carrying a homozygous p.Arg393_Val400dup TYMP variant, which had been previously reported in a single case of classical MNGIE with no documented abnormalities in fat tissue distribution or metabolic parameters [15]. We also review published cases of TYMP-related lipodystrophy.

## 2. Results

### 2.1. Case Description

Our patient is a female of Chinese descent, born to consanguineous parents (second degree cousins). Her father, who had a history of an unspecified cardiac disease, died at the age of 41 from a cause that was not clearly defined. She has two younger siblings (one male, one female), both reportedly healthy. Her mother, the paternal grandparents and the maternal grandmother are alive and reported to be in good health. She was born from the third pregnancy, following one elective termination and one early spontaneous abortion (at approximately 4–5 weeks of gestation). The pregnancy was uneventful, and delivery occurred at term via spontaneous vaginal birth. Birth weight was appropriate for gestational age, and neonatal adaptation was unremarkable. She was exclusively formula-fed due to maternal hypogalactia. Neurodevelopmental milestones were achieved within normal timeframes: she acquired independent sitting at 8 months, autonomous walking at 11 months, and age-appropriate speech development was reported. Menarche occurred at the age of 11, followed by irregular menstrual cycles with episodes of hypermenorrhea.

At the age of 16, the patient underwent her first evaluation at Meyer Children’s Hospital in Florence due to menstrual irregularities and hirsutism. At that time, her height was between the 3rd and 10th percentile, within her target height range, with normal body weight and appropriate pubertal development. Physical examination revealed craniofacial dysmorphisms, including plagiocephaly, a prominent forehead, and a bulbous nose. Acanthosis nigricans was noted in the axillary, cervical, and inguinal regions, along with generalized xerotic skin, hypermelanotic macules, and hirsutism (Ferriman-Gallwey score: 10). A marked reduction in subcutaneous adipose tissue was also observed, particularly in the limbs, with bilateral calf hypertrophy and a sculpted appearance of the abdomen, biceps, and posterior thigh muscles. Neurological examination was unremarkable, although mild executive slowness was noted. Abdominal ultrasound revealed increased hepatic echogenicity, consistent with moderate-to-severe hepatic steatosis. Laboratory tests showed elevated liver enzymes. An oral glucose tolerance test (OGTT) demonstrated 120 min glucose levels consistent with impaired glucose tolerance (7.94 mmol/L), and insulin levels indicative of insulin resistance. The metabolic profile also revealed hypertriglyceridemia. Given the positive family history for cardiac disease, a cardiological evaluation was performed, which showed normal findings. A spinal X-ray revealed a right-convex scoliotic curvature of the thoracolumbar spine, along with cervical spine straightening and a tendency toward inversion.

Urinary organic acid analysis performed during the evaluation revealed elevated urinary excretion of uracil (35 mmol/mol CrU; reference values < 24) and thymine (30 mmol/mol CrU; reference values < 2). This biochemical profile is typically associated with only two metabolic disorders: MNGIE and dihydropyrimidine dehydrogenase (DPD) deficiency. The latter, however, is not associated with lipodystrophy and was clinically incompatible with the patient’s presentation. Based on these findings, targeted analysis of urinary nucleosides was performed, confirming markedly elevated thymidine and deoxyuridine levels (256 and 224 mmol/mol CrU, respectively; normal values ≤ 3), consistent with thymidine phosphorylase deficiency. Considering the association of lipodystrophy with the biochemical profile, the patient was referred to the Obesity and Lipodystrophy Center at the University Hospital of Pisa for further evaluation and genetic confirmation.

**Figure 1:**
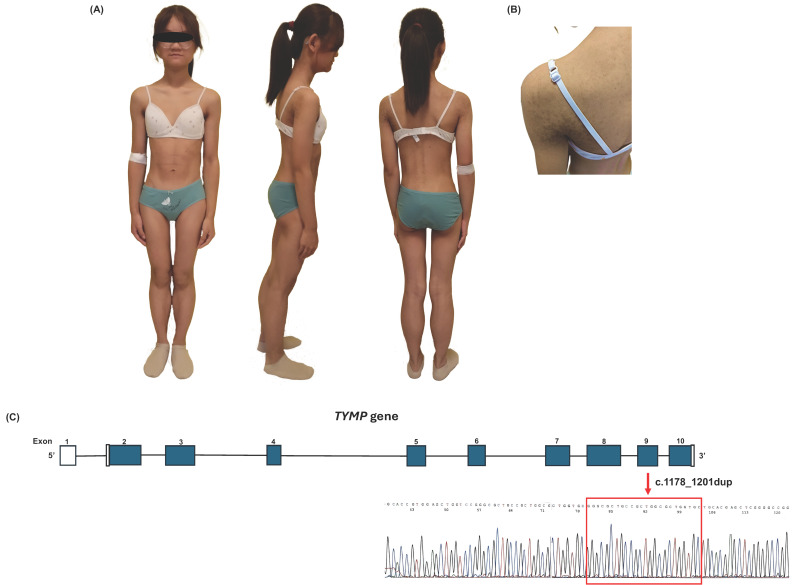
Clinical phenotype and *TYMP* gene analysis in the patient. Anterior, lateral and posterior views of the patient demonstrating generalized loss of subcutaneous fat (**A**), and hypermelanotic macules on the shoulder (**B**). Schematic structure of the *TYMP* gene and Sanger sequencing showing a homozygous c.1178_1201dup variant in the patient. The boxed region corresponds to the duplicated segment (GGGCGCTGCCGCTGGCGCTGGTGC), which results in an in-frame protein duplication p.Arg393_Val400dup (**C**).

On physical examination, she exhibited a generalized reduction in subcutaneous adipose tissue with pseudohypertrophy of the calves and a sculpted appearance of the thighs and abdomen, along with hypermelanotic macules on the shoulders and back (Figure 1A,B). Body height was within normal limits (1.55 m, ~25th percentile), while body mass index was below the normal range (15.4 kg/m^2^, <3rd percentile), according to reference values for Chinese adolescents [16,17] (Table 1). Biochemical blood tests (Table 2) revealed normal levels of fasting glucose, glycated hemoglobin, total cholesterol, low-density lipoprotein (LDL) cholesterol, alanine aminotransferase (ALT), gamma-glutamyl transferase (GGT), and creatine phosphokinase (CPK). A HOMA-IR value of 1.97, falling at the upper limit of the normal range, was consistent with borderline insulin resistance and confirmed the abnormal insulin-glucose dynamics observed during the OGTT, despite fasting insulin levels being within reference limits (Table 2). Hypertriglyceridemia was observed (1.96 mmol/L), accompanied by reduced high-density lipoprotein (HDL) cholesterol levels (0.91 mmol/L). Mild elevations were observed in aminotransferase (ALT), and lactate dehydrogenase (LDH). Endocrinological assessment showed normal thyroid, gonadal, pituitary, and adrenal function. Plasma leptin and high molecular weight (HMW) adiponectin levels were low (2.5 ng/mL and 0.2 mg/L, respectively), consistent with severe adipose tissue deficiency and in line with those usually reported in generalized lipodystrophy [18,19]. Serological screening for both organ-specific and non–organ-specific autoantibodies (including celiac disease–associated antibodies) was negative. Table 1 reports body fat percentage as assessed by dual-energy X-ray absorptiometry (DXA). DXA analysis revealed a reduced fat mass distribution in the upper limbs, lower limbs, and trunk. Abdominal ultrasonography revealed normal liver volume with evidence of mild hepatic steatosis. Transient elastography (FibroScan) showed a controlled attenuation parameter (CAP) of 217 dB/m and liver stiffness measurement (E) of 4.9 kPa. Cardiological evaluation showed no abnormalities on standard 12-lead electrocardiogram (ECG) and transthoracic echocardiography. In particular, the patient had normal biventricular systolic function, with a left ventricular ejection fraction (LVEF) of 70% as assessed by 2D/3D echocardiography. Her biomarker profile was within normal limits, with high-sensitivity troponin T (hs-TnT) at 4 ng/L and undetectable N-terminal pro B-type natriuretic peptide (NT-proBNP).

### 2.2. Genetic Analysis

Given the clinical presentation of generalized lipodystrophy and biochemical findings strongly suggestive of MNGIE, the patient underwent next-generation sequencing (NGS) of genes implicated in congenital forms of lipodystrophy. The analysis identified a homozygous in-frame duplication in the *TYMP* gene. This variant was initially reported in the literature as c.1193_1216dup; p.Arg398_Val405dup, based on transcript ENST00000395681.6 [15]. In accordance with Human Genome Variation Society (HGVS) and College of Medical Genetics and Genomics (ACMG) guidelines, we here refer to the canonical transcript ENST00000252029.8 (NM_001953.5; NP_001944.1), for which the correct nomenclature is c.1178_1201dup; p.Arg393_Val400dup. The variant was confirmed by Sanger sequencing (Figure 1C). Parental segregation analysis revealed that the variant was present in the heterozygous state in the mother, who was asymptomatic. The father was unavailable for testing due to prior death, and genetic analysis of the patient’s siblings is planned for future follow-up.

This variant is not reported in population databases such as gnomAD, dbSNP, or the 1000 Genomes Project, supporting its rarity. It was previously described in a patient with classical MNGIE, in whom in silico analyses using PredictProtein predicted significant structural alterations affecting secondary structure elements, transmembrane domains, and protein–protein interaction regions [15]. In our case, initial evaluation using the JuliaOmix platform classified the variant as a Variant of Uncertain Significance (VUS), based on the ACMG criteria BP4, PM1, and PM2 [20]. However, integration of additional clinical, biochemical, and segregation data provides stronger support for pathogenicity. Specifically, the duplication introduces an in-frame change within a conserved region of the *TYMP* gene that is essential for thymidine phosphorylase activity (PM4), consistent with a loss-of-function mechanism in a gene where such mechanisms are a well-established cause of disease [1,14,21,22]. The patient’s clinical phenotype aligns with known manifestations of TYMP-related disease and includes prominent metabolic features, thus broadening the recognized clinical spectrum [4,14,22,23]. Her biochemical profile, with markedly elevated thymidine and deoxyuridine levels, is highly specific for TYMP deficiency and serum LDH, a known secondary marker of mitochondrial dysfunction, was also elevated (PP4). In addition, segregation analysis confirmed maternal heterozygosity (PP1). When combined with computational evidence from the literature consistently suggesting a deleterious structural effect (PP3) [15], these findings support reclassification of the variant as “likely pathogenic” according to ACMG guidelines (see Table 3 for detailed criteria) and confirm its role in causing MNGIE in this patient. In light of this genetic confirmation, together with the clinical and biochemical context, fully consistent with previously reported TYMP-related cases, the diagnosis of MNGIE was established.

**Figure 2:**
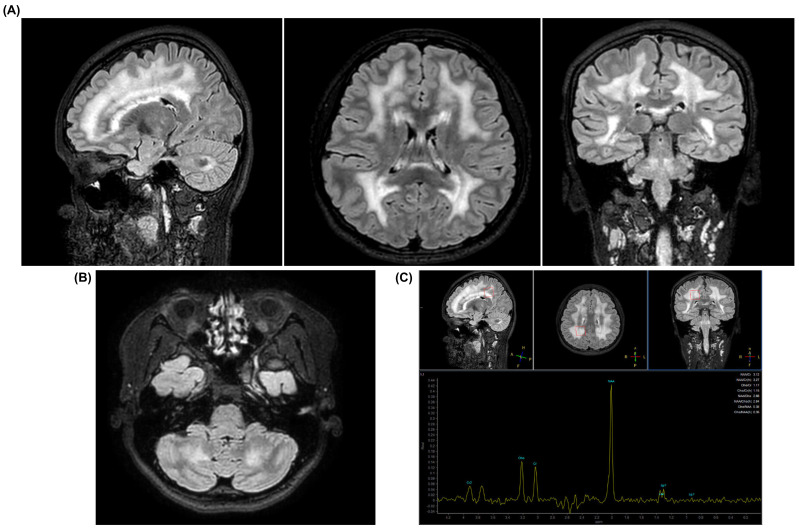
Brain MRI and proton MR spectroscopy findings in the patient. (**A**) Sagittal, axial, and coronal 3D FLAIR images showing diffuse hyperintensities in the periventricular and deep cerebral white matter, more prominent in the frontal and parietal lobes, with involvement of the corpus callosum. (**B**) Axial 3D FLAIR image showing hyperintensity of the deep cerebellar white matter and brainstem involvement. (**C**) Single-voxel proton MR spectrum from the selected ROI in the right parietal white matter (red rectangle in the figure) showing a slight increase in lactate levels.

Following the confirmed diagnosis of MNGIE, the patient underwent a targeted neurological evaluation, including neurophysiological studies, which revealed a moderate peripheral motor polyneuropathy of mixed, predominantly demyelinating, type; mild-to-moderate diffuse myopathic involvement with reduced recruitment patterns in the gastrocnemius muscles bilaterally (in areas of hypertrophy/pseudohypertrophy); and signs of chronic denervation. Brain magnetic resonance imaging (MRI) demonstrated diffuse, bilateral hyperintensities on long TR T2-weighted sequences (FLAIR) involving the periventricular and deep hemispheric white matter, predominantly affecting the frontal and parietal lobes. Signal abnormalities also partially involved the thalami, putamen, splenium of the corpus callosum, as well as, to a lesser extent, the anterior portion of the pons corresponding to the corticospinal and corticonuclear tracts, and the deep cerebellar white matter. These findings are consistent with leukoencephalopathy (Figure 2A,B). In addition, magnetic resonance spectroscopy (MRS) performed in the right parietal region showed a slight increase in lactate levels (Figure 2C). Despite these structural and functional abnormalities of the nervous system, at the most recent neuropsychological assessment the patient’s visuospatial abilities and processing speed were within the normal range for age.

## 3. Discussion

MNGIE is a rare autosomal recessive disorder caused by pathogenic variants in the *TYMP* gene, which encodes thymidine phosphorylase. Loss-of-function mutations in *TYMP* lead to a partial or complete deficiency of enzymatic activity, resulting in systemic accumulation of thymidine and 2′-deoxyuridine [21]. These nucleoside imbalances cause elevated intracellular levels of the corresponding triphosphates, disrupting the physiological homeostasis of deoxyribonucleotides within mitochondria. Consequently, mitochondrial DNA (mtDNA) replication becomes impaired, leading to multiple deletions, somatic point mutations, and mtDNA depletion, ultimately resulting in mitochondrial dysfunction and cellular energy failure [2,3,24,25,26].

This profound bioenergetic deficit particularly compromises tissues with high metabolic demands, such as the nervous and muscular systems, accounting for the characteristic clinical features of MNGIE. The disease is characterized by a progressive, multisystem course and typically presents with a combination of severe gastrointestinal dysmotility, external ophthalmoplegia, peripheral sensorimotor neuropathy (initially affecting the lower limbs), and progressive skeletal muscle weakness [4,27]. Gastrointestinal symptoms, including early satiety, nausea, abdominal pain, diarrhea, and dysphagia, often lead to substantial weight loss and cachexia. These manifestations are largely attributable to autonomic neuropathy involving the enteric nervous system [28]. Some patients may also experience episodes of intestinal pseudo-obstruction, and hepatic involvement, such as steatosis or cirrhosis, has been reported in several cases [14,28]. MRI findings frequently reveal diffuse hyperintensities in the cerebral white matter indicating a leukoencephalopathy [14,29]. The clinical manifestations of MNGIE vary according to the degree of TP deficiency. In patients with the classic form, residual enzymatic activity is typically below 5%, with symptom onset occurring in the second decade of life and life expectancy limited to the fourth decade. In contrast, individuals with partial loss of enzyme function (residual activity around 10–15%) tend to develop symptoms later in adulthood and may survive beyond the fifth decade [30]. An additional pathogenic mechanism contributing to clinical variability is the so-called threshold effect: in MNGIE, the disease phenotype emerges progressively once the proportion of mitochondria harboring mutated mtDNA exceeds a critical threshold, generally estimated at 80-90% of the total mitochondrial population [31,32]. This mechanism likely accounts for both the delayed onset of symptoms and the phenotypic heterogeneity observed among patients. Notably, heterozygous carriers of *TYMP* mutations do not develop the disease. The severity of gastrointestinal involvement is a major determinant of prognosis. Most patients succumb to complications related to intestinal dysfunction, such as cachexia, peritonitis, gastrointestinal bleeding, intestinal rupture, or aspiration pneumonia, with a reported average age at death of approximately 35–37 years [4].

In the current paper, we report the case of a patient presenting with MNGIE and generalized lipodystrophy, in whom we identified a homozygous TYMP variant (p.Arg393_Val400dup), previously reported in a patient with classical MNGIE. In that case, no metabolic or adipose tissue abnormalities were described, apart from a note of marked thinness (BMI 15.06 kg/m^2^) [15]. To date, only three cases of MNGIE associated with a lipodystrophic phenotype have been reported [14]. However, even before these reports, several clinical descriptions of patients with MNGIE included metabolic abnormalities highly suggestive of underlying adipose tissue dysfunction, despite the absence of a formal lipodystrophy diagnosis. These features included hypertriglyceridemia, hepatic steatosis or cirrhosis with hepatomegaly and elevated liver enzymes and early-onset diabetes [22,33,34,35,36,37,38,39,40]. Furthermore, the extreme thinness commonly observed in MNGIE could reflect not only cachexia secondary to gastrointestinal dysfunction, as often assumed, but also primary lipoatrophy due to adipose tissue involvement, either independently or in combination.

Our case closely resembles the three previously reported patients with MNGIE and generalized lipodystrophy, both clinically and biochemically [14] (Table 1 and Table 2). As in those cases, our patient presented with a low BMI, generalized loss of adipose tissue confirmed by DXA, and a consistent metabolic profile characterized by hypertriglyceridemia, hepatic steatosis, insulin resistance or diabetes, and low circulating levels of leptin and adiponectin [14]. Additional shared features, such as acanthosis nigricans, hirsutism, and menstrual irregularities, were also present in all reported cases, reinforcing the phenotypic similarities observed in this subset of patients [14]. Notably, in both our patient and the previously described individuals, the lipodystrophic and metabolic abnormalities appear to be the earliest clinical manifestations, emerging during adolescence (approximately between ages 14 and 18). In contrast, overt neurological and gastrointestinal symptoms, typically central to the MNGIE phenotype, were initially absent in all reported cases, including our patient, who showed no such manifestations at the time of her first clinical evaluation at age 16 [14]. However, as in the other cases, subclinical involvement was already detectable: our patient showed evidence of peripheral motor polyneuropathy on electroneurography, despite lacking overt neurological signs. Additionally, as expected in the context of mitochondrial dysfunction, our patient showed elevated serum lactate dehydrogenase, in line with those reported in previously described cases [14].

Although the clinical presentation is strikingly similar, the TYMP variant identified in our patient (p.Arg393_Val400dup) differs from the mutations previously reported in the three known cases of MNGIE associated with lipodystrophy [14]. This supports the hypothesis that different *TYMP* variants can lead to a common phenotype involving adipose tissue loss and severe metabolic disturbances, particularly during adolescence. At the same time, it underscores the variability in the onset and progression of the neurological and gastrointestinal features typically associated with MNGIE, which may remain subclinical in the early stages of the disease.

The presence of lipodystrophy and metabolic alterations in our patient, as well as in the other reported cases, strongly suggests that adipose tissue dysfunction may represent an integral, albeit underrecognized, component of the MNGIE clinical spectrum. Functional studies using CRISPR-Cas9-mediated knockout of *TYMP* in human adipose-derived stromal cells (ASC) have, for the first time, demonstrated a direct role of thymidine phosphorylase in adipose tissue biology [14]. Loss of TP resulted in a marked impairment of adipocyte differentiation and function, with reduced intracellular lipid accumulation, lower triglyceride content, and decreased expression of adipogenic and mature adipocyte markers. Insulin signaling was also disrupted, even at the pre-adipocyte stage [14]. These findings are in line with the lipoatrophic, and insulin-resistant phenotype observed in our patient and in the previously described cases. Mechanistically, the accumulation of deoxyribonucleosides in TP-deficient cells leads to mitochondrial dysfunction through mtDNA mutations and depletion, ultimately compromising respiratory chain activity and cellular energy production, as previously reported in other affected tissues [41]. In parallel, altered mitochondrial ROS dynamics further impair adipogenesis and promote insulin resistance, supporting a pathophysiological link between TYMP dysfunction and the metabolic alterations characteristic of this subset of patients [14,42,43,44,45].

Current treatment strategies for MNGIE include both temporary and permanent approaches aimed at correcting the systemic biochemical imbalance caused by thymidine phosphorylase deficiency. Temporary treatments, such as hemodialysis (HD), continuous ambulatory peritoneal dialysis (CAPD), erythrocyte-encapsulated TP (EE-TP), and platelet infusions, have shown partial efficacy in reducing toxic nucleoside accumulation and stabilizing the metabolic profile. These options may be used at any age, particularly in patients awaiting definitive therapy [4]. Permanent treatments, including hematopoietic stem cell transplantation (HSCT) and orthotopic liver transplantation (OLT), remain the only interventions capable of restoring sustained TP activity [46,47,48,49,50,51]. Both should be considered as early as possible after diagnosis, particularly in patients at an early disease stage. HSCT is typically preferred in pediatric or young adult patients with minimal hepatic and gastrointestinal involvement and access to a matched donor, while OLT is more appropriate for patients with progressive liver disease [4]. Despite promising results, long-term follow-up indicates that gastrointestinal dysfunction and leukoencephalopathy may not fully reverse, especially when treatment is delayed [46,49,50,52,53]. Early intervention may increase the likelihood of mtDNA recovery in affected tissues, including the gastrointestinal tract, potentially improving clinical outcomes [54]. In light of these considerations, once the diagnosis of MNGIE was confirmed, our patient was promptly referred to the national referral center for MNGIE for comprehensive evaluation and assessment of potential therapeutic options.

## 4. Materials and Methods

### 4.1. Anthropometric Measurements

Height and body weight were measured by standard procedures. Total and segmental body fat in the trunk, upper and lower extremities was evaluated by whole-body dual-energy X-ray absorptiometry (DXA, Lunar Prodigy Advance, PA+41214, GE Medical Systems, Milwaukee, WI, USA). The proportion of fat in specific body districts as well as the whole body was calculated as a percentage of body mass.

### 4.2. Biochemistry and Hormones

All determinations were carried out after at least 12 h of fasting. Leptin was measured by ELISA from Mediagnost, Reutlingen, Germany; HMW adiponectin was detected using Lumipulse^®^ G HMW adiponectin (Fujirebio, Tokyo, 163-0410 Japan). Hormones were determined using automated equipment at the Chemistry and Endocrinology Laboratory at the University Hospital of Pisa, Italy.

### 4.3. Hepatic and Cardiac Evaluation

Abdominal imaging was performed using standard ultrasound, assessing liver size, echotexture, and steatosis. Hepatic fat content and stiffness were further evaluated by transient elastography (FibroScan^®^, Echosens, Paris, France), with results expressed as controlled attenuation parameter (CAP, dB/m) and liver stiffness measurement (LSM, kPa).

Cardiac evaluation included a 12-lead resting electrocardiogram (ECG) and transthoracic echocardiography to assess morphology and systolic function. High-sensitivity troponin T (hs-TnT) and N-terminal pro–B-type natriuretic peptide (NT-proBNP) levels were also measured to assess myocardial injury and function.

### 4.4. Genetic Testing

Genomic DNA was extracted from peripheral blood collected in EDTA tubes. Next-generation sequencing (NGS) was performed using a custom-designed SureSelectXT panel (Agilent Technologies, Santa Clara, CA, USA) targeting the coding exons and exon–intron boundaries of genes associated with congenital lipodystrophy and related syndromes (*ADRA2A*, *AGPAT2*, *ALDH18A1*, *AIRE*, *AKT2*, *BANF1*, *BLM*, *BSCL2*, *BUD13*, *CAV1*, *CAVIN1*, *CIDEC*, *DYRK1B*, *DDR2*, *EPHX1*, *ERCC3*, *ERCC6*, *ERCC8*, *FBN1*, *INSR*, *KCNJ6*, *LEMD2*, *LIPE*, *LMF1*, *LMNA*, *LMNB2*, *MDM2*, *MFN2*, *MTX2*, *NSMCE2*, *OPA3*, *OTULIN*, *PCNT*, *PCYT1A*, *PDGFRB*, *PIK3R1*, *PLAAT3*, *PLIN1*, *POC1A*, *POLD1*, *POLR3A*, *POLR3B*, *PPARG*, *PTPN11*, *POLR3GL*, *POMP*, *PSMA3*, *PSMB4*, *PSMB8*, *PSMB9*, *PSMG2*, *PYCR1*, *SLC25A24*, *SLC29A3*, *SPRTN*, *TYMP*, *VIM*, *WRN*, *ZMPSTE24*). Sequencing was conducted on the Illumina MiSeqDx platform, and reads were aligned to the GRCh38/hg38 human genome reference. Data analysis was performed using SureCall software (Agilent Technologies, Santa Clara, CA, USA), and variant annotation was based on multiple databases, including Franklin by QIAGEN (originally developed by Genoox Ltd.; QIAGEN, Hilden, Germany), Ensembl (European Bioinformatics Institute, Hinxton, Cambridge, UK), ClinVar (National Center for Biotechnology Information, Bethesda, MD, USA), Varsome (Saphetor SA, Lausanne, Switzerland), and dbSNP (National Center for Biotechnology Information, Bethesda, MD, USA). Variant classification was performed using the JuliaOmix platform (GenomeUp srl, Rome, Italy) according to the ACMG guidelines. All online tools and databases were accessed on 27 January 2025.

Genotype was confirmed by Sanger sequencing. Specific primers were designed using Primer 3 (http://primer3.ut.ee/ Primer3web version 4.1.0, accessed on 3 December 2024) to amplify the exon 9 from genomic DNA, isolated from whole blood. PCR was performed using PCR Master Mix (AmpliTaq Gold^TM^ 360 Master Mix, Thermo Fisher Scientific, Waltham, MA, USA), with an annealing temperature of 55 °C. After purification with ExoProStar (GE Healthcare UK Limited, Amersham, UK), the PCR products were sequenced using Applied Biosystem 3500XL (Thermo Fisher Scientific, Waltham, MA, USA).

## 5. Conclusions

We describe a case of MNGIE presenting with a generalized lipodystrophy and metabolic derangements in a patient harboring a homozygous *TYMP* variant (c.1178_1201dup; p.Arg393_Val400dup), previously reported in classical MNGIE without documented adipose tissue dysfunction or metabolic manifestations. This case reinforces the concept that adipose tissue involvement may be an integral, yet underrecognized, feature of the disease. Despite genotypic differences, the remarkable clinical and biochemical similarities with previously described cases of MNGIE presenting with lipodystrophy support the hypothesis of a shared pathophysiological mechanism, likely driven by early mitochondrial dysfunction in adipose tissue. The present report contributes to broadening the genotypic and phenotypic spectrum of TYMP-related disease and underscores the need to consider possible TYMP involvement in the differential diagnosis of atypical or syndromic lipodystrophies, particularly in the presence of findings suggestive of mitochondrial dysfunction. Early diagnosis is crucial, as it allows for timely referral to specialized centers for comprehensive evaluation and consideration of therapeutic options, such as HSCT or liver transplantation. These interventions may alter disease progression if performed before irreversible organ damage occurs. Further studies are warranted to elucidate the molecular mechanisms linking TYMP dysfunction to adipose and metabolic alterations, and to better define the natural history, prognosis, and treatment responsiveness of this emerging phenotypic subset.

## Figures and Tables

**Table 1:** Clinical Features of our Index Patient compared to Previously Reported Cases of Lipodystrophy Associated with *TYMP* Variants.

	Patient 1 (Index Case)	Patient 2	Patient 3	Patient 4
	Current Study	[14]	[14]	[14]
Sex	F	F	M	F
TYMP variant	c.1178_1201dup, HO	c.647-1G>A, HO	c.647-1G>A, HO	c.392C>T, HO
Age at report (y)	16	Deceased at the age of 24	27	20
Height (m)	1.55	1.63	1.70	1.60
Weight (kg)	37	34	53	37.5
BMI (kg/m^2^)	15.4	12.8	18.3	14.6
Lipodystrophy	Generalized	Generalized	Generalized	Generalized
Hypermuscular appearance	Yes	No	Yes	Yes
Acanthosis nigricans	Yes	Yes	Yes	Yes
Liver steatosis	Yes	Yes	Yes	Yes
Hypertriglyceridemia	Yes	Yes	Yes	Yes
Diabetes/IR	Yes	Yes	No	Yes
Hirsutism	Yes	Yes	NA	Yes
Menstrual abnormalities	Yes, hypermenorrhea	Yes, amenorrhea	NA	Yes, amenorrhea
Neurological signs	Yes, motor peripheral neuropathy, leukoencephalopathy	Yes, demyelinating sensory motor peripheral neuropathy, electromyogram abnormalities, leukoencephalopathy, ptosis, muscular atrophy	Yes, leukoencephalopathy	Yes, demyelinating sensory motor peripheral neuropathy, electromyogram abnormalities, leukoencephalopathy
Gastrointestinal signs	No	Yes, gastroparesis, abdominal pain	No	Yes, gastroparesis, abdominal pain, diarrhea
Whole body fat (%)-DXA	16.9	9.6	8.4	NR
Arm fat (%)-DXA	21.4	9.9	6.95	NR
Leg fat (%)-DXA	19.4	8.7	5.5	NR
Truncal fat (%)-DXA	12.3	8.1	8.5	NR

Abbreviations: HO, homozygous; IR, insulin resistance; NA, not applicable; NR, not reported, DXA, dual-energy X-ray absorptiometry.

**Table 2:** Biochemical tests of our Index Patient compared to Previously Reported Cases of Lipodystrophy Associated with *TYMP* Variants.

	Patient 1 (Index Case)	Patient 2	Patient 3	Patient 4
	Current Study	[14]	[14]	[14]
Fasting glucose, mmol/L	3.11 (3.3–5.5)	5.2 (4.1–6.1)	4.2 (4.1–6.1)	14.2 (4.1–6.1)
2h-OGTT glucose, mmol/L	7.94 (≤7.8)	13 (≤7.8)	10.9 (≤7.8)	NR
Fasting insulin, pmol/L	37 (<160)	358.3 (<70)	519.4 (<70)	530.6 (<70)
HbA1c, (%)	5.2 (<6)	5 (<6)	4.9 (<6)	8.4 (<6)
AST, IU/L	27 (<40)	142 (<40)	100 (<40)	50 (<40)
ALT, IU/L	45 (<40)	52 (<40)	134 (<40)	40 (<40)
γGT, IU/L	33 (<36)	77 (8–44)	65 (8–44)	142 (8–44)
Total cholesterol, mmol/L	3.72 (<5.17)	NR	NR	NR
LDL-cholesterol, mmol/L	2.17 (<2.97)	NR	NR	NR
HDL-cholesterol, mmol/L	0.90 (>1.16)	0.77 (>1)	0.65 (>1)	0.33 (>1)
Triglycerides, mmol/L	1.95 (<1.69)	7.6 (<1.70)	2.2 (<1.70)	28.1 (<1.70)
LDH, IU/L	228 (135–214)	325 (120–246)	256 (120–246)	212 (98–192)
Leptin, ng/mL	2.5	1.9	0.5	0.53
Adiponectin, mg/L	0.2	<0.01	0.37	NR

Abbreviations: OGTT, oral glucose tolerance test; HbA1c, hemoglobin A1c; AST, aspartate aminotransferase; ALT, alanine aminotransferase; γGT, gamma glutamyl transferase; NR, not reported; LDL-cholesterol, low-density lipoprotein cholesterol; HDL-cholesterol, high-density lipoprotein cholesterol; LDH, lactate dehydrogenase. Values in brackets indicate the normal range of reported values.

**Table 3:** ACMG criteria applied to the *TYMP* c.1178_1201dup; p.Arg393_Val400dup variant.

Criterion	Description (ACMG/AMP 2015)	Evidence in Our Case
PM2	Absent from population databases	Variant not reported in gnomAD, dbSNP, or 1000 Genomes Project
PM4	Protein length changes due to in-frame insertion/duplication in a non-repeat region	In-frame duplication within a conserved C-terminal region critical for thymidine phosphorylase function
PP1	Co-segregation with disease in multiple affected family members in a gene definitively known to cause the disease	Variant found in heterozygous state in the mother (asymptomatic carrier); paternal testing not possible
PP3	Multiple computational predictions support a deleterious effect	In silico analyses (PredictProtein in prior report [15]) indicate structural disruption of thymidine phosphorylase
PP4	Patient’s phenotype highly specific for a disease with a single genetic etiology	Clinical and biochemical features fully consistent with TYMP-related disease

## Data Availability

The original contributions presented in this study are included in the article material. Further inquiries can be directed to the corresponding author.
